# Study of the Associations between Color Doppler Ultrasound Grading of Hyperthyroidism and Biochemical Data on Thyroid Function

**DOI:** 10.1155/2022/9743654

**Published:** 2022-07-30

**Authors:** Lingyun Zhang, Jie Li, Suzhen Zhang, Chen Su, Zengcun Su, Yuezhong Zhang, Yonghao Gai, Shanshan Shao, Jianzhi Li, Guoquan Zhang

**Affiliations:** ^1^Department of Ultrasonography, Shandong Provincial Hospital, Shandong First Medical University, Jinan, China; ^2^Department of Outpatient, Outpatient of Jinan Municipal People's Government, Jinan, China; ^3^Shandong Provincial Key Laboratory of Endocrinology and Lipid Metabolism, Jinan, China; ^4^Department of Ultrasonography, Shandong Public Health Clinical Center, Jinan, China

## Abstract

**Objective:**

The main study objective was to investigate the correlation between the color Doppler ultrasound grading of hyperthyroidism and the biochemical data of thyroid function.

**Methods:**

Seventy-six patients were diagnosed with hyperthyroidism based on clinical and laboratory data at our hospital. The patients were examined using color Doppler ultrasound and laboratory investigations before starting ^131^I treatment. First, patients were divided into two groups based on the blood flow distribution determined by ultrasound. If the blood flow signal in the parenchyma was scattered and thinned, with dispersive points and discontinuous streaky distribution, the blood flow distribution area in the sample frame was less than or equal to 1/2 of the sample frame area and was judged to be level 1. If the parenchyma was filled with diffuse blood flow signals or if most areas had depicted rich blood flow distribution when the area of blood flow distribution in the sampling frame was greater than 1/2 of the sampling frame area, it was judged to be level 2. Then, the correlations between color Doppler ultrasound grading and biochemical data of thyroid function were analyzed. The indices included FT3, FT4, TSH, anti-TG, anti-TPO, and TRAb. Parameters of thyroid homeostasis, including thyroid's secretory capacity (SPINA-GT), the total deiodinase activity (SPINA-GD), Jostel's TSH index, and the thyrotroph thyroid hormone sensitivity index (TTSI), were calculated and compared.

**Results:**

Correlations were noted between color Doppler ultrasound grading and FT3, FT4, TRAb, SPINA-GT, TSHI, and TTSI. Moreover, FT3, FT4, TRAb, SPINA-GT, TSHI, and TTSI were higher in level 2 patients compared with level 1 patients.

**Conclusion:**

Correlations were noted between color Doppler ultrasound grading and biochemical data of thyroid function.

## 1. Introduction

Hyperthyroidism is a common autoimmune thyroid disease. Modern ultrasound, including high-resolution ultrasound, harmonic imaging, elastography, and contrast-enhanced ultrasound, is increasingly recommended as the first-line investigative tool in the differential diagnosis of hyperthyroidism. Color Doppler ultrasound is a powerful technique that combines the grayscale view of conventional sonography with the colored display of blood flow. The method provides an evaluation of the vascular patterns of the thyroid gland, indirectly measuring thyroid function [1]. Previous studies have revealed that the thyroid sonogram changes in patients with hyperthyroidism include thyroid enlargement, uneven echo, and “thyroid inferno” (a pulsatile pattern of intrathyroidal flow in multiple areas both in diastole and in systole described by Ralls et al. in 1988 and termed “inferno.” [2]) in color Doppler flow imaging. However, in clinical practice, thyroid sonograms of some patients with hyperthyroidism may also reveal other characteristics, such as difference in blood flow signals in the thyroid. Whether these differences are related to indicators of thyroid function remains unknown. The correlation between sonographic thyroid patterns and thyroid function tests is controversial [3, 4]. Therefore, this study aimed to retrospectively review color Doppler ultrasound grading and biochemical data from hyperthyroidism patients.

## 2. Materials and Methods

### 2.1. Patients

Seventy-six patients from a retrospectively collected database of patients with hyperthyroidism referred to the endocrinology unit were hospitalized between January 2016 and May 2018. All of the subjects underwent thyroid ultrasound and thyroid function tests. The diagnosis of hyperthyroidism was based on goiter, clinical signs of thyrotoxicosis (such as tremors, high pulse rate, diaphoresis, and diarrhea), ophthalmopathy (when present), and the indices of thyroid function. These patients had not been under antithyroid drug treatment for at least two weeks.

Patients with toxic multinodular goiters were not included in the study. Patients with a history of thyroid gland surgery or radioactive iodine treatment were also excluded. Patients in this group did not have a history of chronic drug use.

### 2.2. Ultrasound Examination

The operator performing the ultrasound was blinded to the results of the laboratory investigation. The examination was performed by the same operator utilizing the same ultrasound system (HI VISION ASCENDUS, Japan) with a linear-array transducer (5–13 MHz). The patients were examined in a supine position with an extended neck, and a cushion was placed under their shoulders.

The largest thyroid section was found by transverse and longitudinal scanning. Then, color Doppler flow imaging was performed. The pulse repetition frequency was adjusted to 6.5 kHz, and the Doppler gray frequency was adjusted to 13 MHz. The color gain was adjusted to prevent artifacts. The patient was asked to hold his breath and keep his head still to observe the blood flow without colored overflow, and the distribution of blood flow in the thyroid was recorded.

### 2.3. Indices of Thyroid Function

Chemiluminescent methods (Cobas E601, Roche, Basel, Switzerland) were employed to determine thyroid function (FT3, FT4, and TSH) for the 76 patients. Cobas E601 (Roche, Basel, Switzerland) and ADVIA Centaur XP (Siemens, Germany) were used to determine thyroid autoantibodies (anti-TG, anti-TPO, and TRAb) in the 76 patients. The laboratory reference ranges were as follows: 3.1–6.8 pmol/L for FT3, 12–22 pmol/L for FT4, 0.27–4.2 *u* IU/L for TSH, 0–115 IU/L for anti-TG, 0–34 IU/L for anti-TPO, and 0–1.58 for TRAb.

Two structure parameters of thyroid homeostasis, including the sum activity of step-up deiodinases (SPINA-GD) and thyroid secretory capacity (SPINA-GT) and two pituitary thyrotropic function indices, including Jostel's TSH index (TSHI) and the thyrotroph thyroid hormone resistance index (TTSI), were calculated on the basis of circulating levels of thyrotropin, free thyroxine, and free triiodothyronine as previously described using the online freely available SPINA Thyr 4.2.0.861 for Mac Universal software.

### 2.4. Color Doppler Ultrasound Grading of Hyperthyroidism

**Figure 1:**
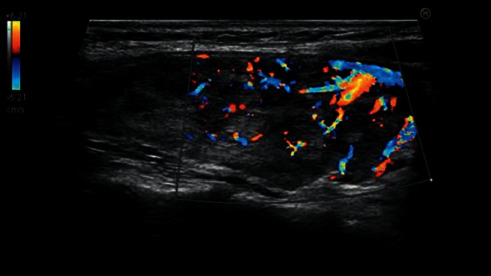
Color Doppler ultrasound grading of hyperthyroidism level 1.

**Figure 2:**
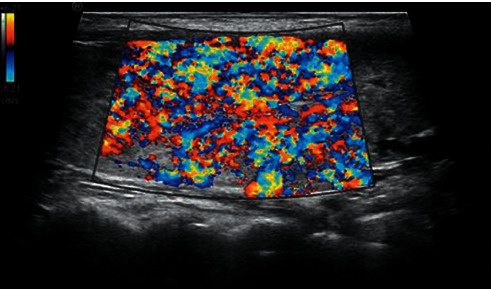
Color Doppler ultrasound grading of hyperthyroidism level 2.

Color Doppler ultrasound was used to determine the richness of the blood flow signal inside the thyroid parenchyma in longitudinal sections. If the blood flow signal in the parenchyma was scattered and thinned, with dispersive points and discontinuous streaky distribution, the blood flow distribution area in the sample frame was less than or equal to 1/2 of the sample frame area and was judged to be level 1 (Figure 1). If the parenchyma was filled with diffuse blood flow signals or if most areas had depicted rich blood flow distribution when the area of blood flow distribution in the sampling frame was greater than 1/2 of the sampling frame area, it was judged to be level 2 (Figure 2).

### 2.5. Statistical Analysis

Statistical analyses were performed using SPSS version 24 (IBM Corp, Armonk, NY, USA). The Kolmogorov–Smirnov test and Shapiro–Wilk test were used to evaluate the normal distribution of continuous variables. Categorical variables were described as frequencies (%) and tested with the *X*^2^ test as appropriate. For data with a nonnormal distribution, medians with an interquartile range (IQR) are reported, and these data were compared using the Mann–Whitney *U* test. A Spearman correlation analysis was performed between the color Doppler ultrasound grading of hyperthyroidism and the biochemical data of thyroid function. A *p* value < 0.05 was considered statistically significant.

## 3. Results

### 3.1. Baseline Clinical Characteristics of Study Subjects

Clinical characteristics of the two levels were summarized in (Table 1). Our results showed that the BMI for the two levels were 22.30 ± 3.80 and 22.41 ± 3.50 kg/m^2^, respectively; the course of hyperthyroidism for the two levels were 1.00 (0.75, 6.75) and 2.50 (0.44, 6.75) years, respectively. No significant differences in sex, age, BMI, and course were noted between level 1 and level 2 patients with hyperthyroidism (*P* > 0.05).

### 3.2. Comparison between the Color Doppler Ultrasound Grading of Hyperthyroidism and the Biochemical Data of Thyroid Function

Statistical difference was observed between the two levels in FT3, FT4, TRAb, SPINA-GT, TSHI, and TTSI. The values of FT3, FT4, TRAb, SPINA-GT, TSHI, and TTSI were higher in level 2 than level 1 (Table 2) (*P* < 0.05).

### 3.3. Relationship between the Color Doppler Ultrasound Grading of Hyperthyroidism and the Biochemical Data of Thyroid Function

There were correlations between the color Doppler ultrasound grading and FT3, FT4, TRAb, SPINA-GT, TSHI, and TTSI. FT3 was positively correlated with blood flow grading in hyperthyroidism patients (*r*= 0.411, *P* ≤ 0.001) using Pearson correlation analysis. FT4 was positively correlated with blood flow grading in hyperthyroidism patients (*r*= 0.399, *P* ≤ 0.001). TRAb was positively associated with blood flow grading in hyperthyroidism patients (*r*= 0.292, *P*=0.011). SPINA-GT was positively correlated with blood flow grading in hyperthyroidism patients (*r*= 0.242, *P*=0.035); TSHI was positively correlated with blood flow grading in hyperthyroidism patients (*r*= 0.369, *P*=0.011). In addition, TTSI was negatively correlated with blood flow grading in hyperthyroidism patients (*r*= −0.237, *P*=0.040) (Table 3).

## 4. Discussion

Hypervascularization appears to be an important radiographic feature in patients with Graves' disease at the onset [5]. Hyperthyroidism is a chronic inflammatory condition that shares similar ultrasonographic features. On average, the blood flow signal in the gland was significantly increased in patients who presented with goiters, heterogeneity, and hypoechoic. Previous studies have described increased blood flow signals in glands as “thyroid inferno” based on the influence of nodules. However, these visualizations do not characterize the thyroid flow in hyperthyroidism patients with different blood flow characteristics during color Doppler ultrasound. Therefore, we qualitatively analyzed the thyroid blood flow in patients with hyperthyroidism in this study. If the blood flow signal in the parenchyma was scattered and thin with dispersive points and discontinuous streaky distribution or the blood flow distribution area in the sample frame was less than or equal to 1/2 of the sample frame area, the patient was judged to be level 1. However, if the parenchyma was filled with diffuse blood flow signals (thyroid inferno) or if most areas had depicted rich blood flow distribution, when the area of blood flow distribution in the sampling frame was greater than 1/2 of the sampling frame area, it was adjudged to be level 2. Castagnone et al. established the usefulness of color Doppler sonography in Graves' disease patients with active hyperthyroidism, enlarged thyroid, and greater intrathyroidal vascularization compared to controls and those patients not receiving antithyroid drugs [6].

Color Doppler ultrasound can be used to visualize the degree of goiter, echo heterogeneity, and blood flow distribution. Notably, the difference in the abundance of blood flow signals in the thyroid parenchyma, which has different clinical and pathophysiological associations, can also be determined. Ralls et al. demonstrated the increased thyroid vascularization of 15 patients with Graves' disease for the first time. The condition was termed the “thyroid inferno” with patterns showing “a pulsatile pattern of intrathyroidal flow in multiple areas, both in diastole and in systole” [2]. This study aimed to retrospectively review color Doppler ultrasound grading and biochemical data of hyperthyroidism disease patients. In the present study, we found a significant association between the color Doppler ultrasound grading of hyperthyroidism and FT3, FT4, and TRAb levels. FT4 and TRAb levels were consistent with those reported by Vita *R* et al. However, the FT3 levels were in contrast with those reported by Vita et al. [5]. The association between blood flow grading and FT3 was 0.411, which was more significant than the association with FT4 (0.399, *p* < 0.05), indicating considerable relatedness of the thyroid blood flow grading with the thyroid function index FT3 compared with FT4. The results are consistent with the physiological basis that FT3 has strong and fast activity in vasodilatation and increasing blood flow in thyroid hormones. FT3 could increase blood flow by altering tissue oxygen consumption (thermogenesis), vascular resistance, blood volume, cardiac contractility, heart rate, and dilation of small arteries of peripheral circulatory resistance to impact the vascular bed [7].

Antibodies against TSH receptors (TRAb) are responsible for the uncontrolled stimulation of the thyroid. The overproduction of thyroid hormones induces growth and increases the blood flow of the gland [3, 8–14]. In hyperthyroidism patients, TSHR autoantibodies (TRAb) stimulate the thyroid after binding to TSHR by increasing the production of intracellular cyclic AMP. The antibody-receptor complex promotes the activation of intracellular cyclic AMP with subsequent thyrocyte hyperplasia (causing gland enlargement) and increased vascularity [15], which is consistent with the findings of the present study. Moreover, TRAb could affect the distribution of blood flow within the thyroid of hyperthyroidism patients. Thyroid vascularization depends on thyroid stimulation by TSH or TRAb. [16]. In the present study, we observed that TRAb values affect the thyroid blood flow distribution in hyperthyroidism patients. However, no significant differences in TSH were noted between level 1 and level 2 patients with hyperthyroidism.

Combining hormone levels with model-based calculations delivers structure parameters of thyroid homeostasis, which may in certain conditions add valuable information for clinical research and differential diagnosis of thyroid disorders. We calculated the correlations between structural parameters of thyroid homeostasis and color Doppler ultrasound grading of hyperthyroidism. The results showed that SPINA-GT and TSHI was positively correlated with blood flow grading in hyperthyroidism patients. In addition, TTSI was negatively correlated with blood flow grading in hyperthyroidism patients.

The systemic and localized hemodynamic alterations within the thyroid interact and contribute to each other. Thus, hyperthyroidism patients exhibit characteristic systemic and local clinical signs and symptoms. Because of the increased blood flow within the thyroid in patients with hyperthyroidism, many investigators have also undergone quantitative measurements of ultrasound blood flow [3, 4, 17]. However, the data vary widely and lack comparability and a practical basis for clinical diagnosis. The main reason is that the quantitative color Doppler technique involves carefully adjusting the ultrasound system to prevent human interference, which represents a challenge for the operator. In addition, given that the thyroid arteries are relatively small vessels with significant variability, it is difficult to obtain reliable, accurate, and ideal blood flow/acoustic beam angles guided by either two-dimensional grayscale ultrasound or color Doppler flow visualization. Thus, these features will significantly impact the measurement of flow and flow velocity. However, quantitative Doppler techniques require careful adjustments of the ultrasound system to prevent artifacts, and these adjustments can be challenging to perform in certain patients. In addition, these methods have not been standardized (for instance, it is often debated which artery needs sampling). National and international guidelines have not included quantitative techniques for diagnosing and managing hyperthyroidism [4, 18, 19].

All the above points are the main reasons why quantitative measurement indices are difficult to widely accept in clinical practice. In this study, the abundance of parenchymal blood flow in the thyroid gland was qualitatively evaluated using the color Doppler ultrasound technique in hyperthyroidism patients. In addition, we analyzed the association between color Doppler ultrasound data and biochemical data of thyroid function. We found that higher levels of FT3, FT4, TRAb, SPINA-GT, TSHI, and TTSI are associated with richer parenchymal blood flow in the thyroid gland, which would provide a convenient and rapid ultrasound imaging tool for functional evaluation in the clinic. Moreover, the color Doppler ultrasound technique uses fewer adjustment parameters with readily available results. Its qualitative examination counteracts the limitation of previous quantitative studies on thyroid parenchymal blood flow.

The current study also has some limitations, such as the small sample size. Moreover, the method to assess vascularization is very subjective and may represent a major bias. In addition, various manifestations of thyroid blood flow distribution exist. Our study only evaluated two groups due to clinical practicality. However, color Doppler ultrasound is relatively mature, and operators in the same hospital can develop fixed technical means and diagnostic guidelines after a certain period of clinical practice.

## 5. Conclusions

Our study found a significant association between the qualitative examination characteristics of thyroid ultrasound, particularly the increased blood flow signal, and the biochemical data of thyroid function, providing a valuable indicator for clinical judgment and treatment evaluation.

## Figures and Tables

**Table 1:** Baseline clinical characteristics of the 76 subjects according to color Doppler ultrasound grading.

	Level 1 (*n*= 12)	Level 2 (*n*= 64)	*P*
Sex (male),*n* (%)	3 (25.0)	13 (20.3)	0.715
Age (years)	32.00 (28.25, 50.25)	34.00 (28.00, 44.50)	0.909
BMI (kg/m^2^)	22.30 ± 3.80	22.41 ± 3.50	0.917
Course (years)	1.00 (0.75, 6.75)	2.50 (0.44, 6.75)	0.814

**Table 2:** Mann–Whitney *U* test between the color Doppler ultrasound grading of hyperthyroidism and the biochemical data of thyroid function.

The biochemical data of thyroid function	Level 1 (*n*= 12) M (Q25, Q75)	Level 2 (*n*= 64) M (Q25, Q75)	*Z*	*P*
FT3 (pmol/L)	8.95 (6.12, 12.20)	24.66 (13.31, 30.80)	−3.907	≤0.001
FT4 (pmol/L)	28.64 (18.91, 35.67)	54.58 (33.31, 83.29)	−3.777	≤0.001
TSH (*u* IU/ml)	0.005 (0.004, 0.007)	0.005 (0.005, 0.008)	−0.426	0.670
Anti-TG (IU/ml)	89.45 (15.00, 319.98)	148.15 (32.00, 500.00)	−1.369	0.171
Anti-TPO (IU/ml)	265.15 (65.44, 1207.95)	222.00 (41.08, 848.85)	−0.315	0.753
TRAb (IU/l)	4.50 (1.24, 6.19)	9.01 (4.33, 20.00)	−2.993	0.003
SPINA-gt (pmol/s)	1090.90 (672.13, 1548.37)	2248.87 (1013.36, 3414.35)	−2.636	0.008
SPINA-gd (nmol/s)	34.02 (27.84, 37.27)	36.72 (31.69, 41.94)	−1.524	0.127
TSHI	−2.23 (−1.20, 0.25)	2.15 (−0.18, 5.52)	−3.441	0.001
TTSI	0.79 (0.34, 1.20)	1.33 (0.84, 2.20)	−2.273	0.023

**Table 3:** Correlation analysis between the color Doppler ultrasound grading of hyperthyroidism and the biochemical data of thyroid function.

The biochemical data of thyroid function	Color Doppler ultrasound grading	
	*r*	*P*
FT3 (pmol/L)	0.411	≤0.001
FT4 (pmol/L)	0.399	≤0.001
TSH (*u* IU/ml)	−0.223	0.053
Anti-TG (IU/ml)	0.127	0.275
Anti-TPO (IU/ml)	−0.039	0.740
TRAb (IU/l)	0.292	0.011
SPINA-gt (pmol/s)	0.242	0.035
SPINA-gd (nmol/s)	0.154	0.185
TSHI	0.369	0.001
TTSI	−0.237	0.040

## Data Availability

Data can be obtained from the corresponding author on request.
